# Improving Phage-Biofilm In Vitro Experimentation

**DOI:** 10.3390/v13061175

**Published:** 2021-06-19

**Authors:** Stephen T. Abedon, Katarzyna M. Danis-Wlodarczyk, Daniel J. Wozniak, Matthew B. Sullivan

**Affiliations:** 1 Department of Microbiology, The Ohio State University, Columbus, OH 43210, USA; wozniak.1@osu.edu (D.J.W.); sullivan.948@osu.edu (M.B.S.); 2 Department of Microbial Infection and Immunity, The Ohio State University, Columbus, OH 43210, USA; danis-wlodarczyk.1@osu.edu; 3 Department of Civil, Environmental and Geodetic Engineering, The Ohio State University, Columbus, OH 43210, USA

**Keywords:** bacteriophage therapy, biofilm, biofouling, biocontrol, biological control, chronic infection, microcolony, phage ecology, phage therapy

## Abstract

Bacteriophages or phages, the viruses of bacteria, are abundant components of most ecosystems, including those where bacteria predominantly occupy biofilm niches. Understanding the phage impact on bacterial biofilms therefore can be crucial toward understanding both phage and bacterial ecology. Here, we take a critical look at the study of bacteriophage interactions with bacterial biofilms as carried out in vitro, since these studies serve as bases of our ecological and therapeutic understanding of phage impacts on biofilms. We suggest that phage-biofilm in vitro experiments often may be improved in terms of both design and interpretation. Specific issues discussed include (a) not distinguishing control of new biofilm growth from removal of existing biofilm, (b) inadequate descriptions of phage titers, (c) artificially small overlying fluid volumes, (d) limited explorations of treatment dosing and duration, (e) only end-point rather than kinetic analyses, (f) importance of distinguishing phage enzymatic from phage bacteriolytic anti-biofilm activities, (g) limitations of biofilm biomass determinations, (h) free-phage interference with viable-count determinations, and (i) importance of experimental conditions. Toward bettering understanding of the ecology of bacteriophage-biofilm interactions, and of phage-mediated biofilm disruption, we discuss here these various issues as well as provide tips toward improving experiments and their reporting.

## 1. Introduction

Bacteria have a propensity to bind to surfaces and to each other. In the process, they create multicelled, spatially structured communities that are embedded in a self-produced polymer matrix [1,2,3,4]. These biofilms occur commonly in natural, industrial, and medical settings [5,6,7,8]. Biofilms are often also correlated with the pathogenesis of bacterial diseases [9,10,11,12,13,14,15], play roles in the evolution of antibiotic resistance [16,17,18,19,20], and can protect sensitive bacteria from antimicrobial agents [13,21,22,23,24,25]. Here, we focus on improving in vitro approaches to understanding the ecology of bacteriophage-biofilm interactions, including, as can result in phage-mediated biofilm disruption.

Phages, known more formally as bacteriophages, are the viruses of bacteria. Use of phages to treat undesirable bacterial colonization and infections can be described as phage therapy [26], or more generally as phage-mediated biocontrol [27,28,29]. Phages have a history of successful treatment of chronic bacterial infections [30,31], including infections associated with wounds [31,32,33,34,35,36] or lungs [37,38,39,40,41,42,43]. These infections often have biofilm components that can severely limit and complicate traditional treatment approaches and resulting outcomes [10,11,12,13]. Consequently, there has been a growing interest in phage anti-biofilm properties and phage-biofilm interactions [8,33,44,45,46,47,48,49,50,51,52,53,54,55,56,57,58].

While we are appreciative as well as humbled by the extent and diversity of the growing literature exploring phage-biofilm in vitro interactions, this emergent area of research includes practices that may limit the utility of many studies. Toward helping to remedy this situation, we consider here a number of approaches to phage-biofilm in vitro experimentation that we suggest may be improved upon (Box 1 and Section 3).

Box 1 **Toward improving phage-biofilm in vitro experimentation: issues and recommendations ^1^.**

1. Distinguishing biofilm control from biofilm removal: importance of zero-time-point determinations
  - Measure biofilm properties (CFUs, thickness, etc.) just prior to phage treatment, i.e., at time zero
  - Following treatments, compare biofilm properties to *both* zero-point and mock-treatment controls
2. Knowledge of phage titers is needed for the interpretation and reproducibility of experiments
  - Explicitly report titers of each phage applied, including, if possible, the expected resulting in situ titers
  - MOI-based dosing, if used, should unambiguously report CFU concentrations as measured at time zero
3. There are conceptual problems with per-area rather than titer-based dosing
  - Report dosing as phage titers, i.e., PFUs/mL of phage-containing volumes applied to surfaces
  - Report per-area dosing also as volumes applied, e.g., 100 μL/cm^2^
4. Overlying fluid small-volume effects
  - If dosing with lower titers, e.g., <<10^8^ PFUs/mL, then measure planktonic CFUs and PFUs over time
  - Or use sufficient titers that substantial in situ phage propagation is not necessary, e.g., ≥10^8^ PFUs/mL
5. Dosing with insufficient phage titers?
  - If biofilm reductions are insufficient, repeat experiments with higher phage titers and/or multiple dosing
  - Consider dosing with maximum achievable titers if biofilm reductions remain inadequate
6. Insufficient numbers of time points?
  - “Good laboratory practices include… determination of time courses…” [59]
  - If possible, repeat experiments using alternative treatment durations, e.g., both 12 and 24 h
7. Enzymatic biofilm matrix disruption
  - Biofilm matrix degradation by EPS depolymerases can impact biofilms even without active phage infection
  - Measure potential phage EPS depolymerase activity against all experimentally targeted bacterial strains
8. Limitations of biofilm biomass determinations
  - Quantifying biofilm presence using solely biomass determinations can be both inaccurate and imprecise
  - If possible, characterize biofilm presence using additional methods, such as CFU counts
9. Colony count complications (exposure to free phages during CFU enumeration)
  - Disrupt biofilms within the largest volumes that are easily achieved and worked with
  - Disrupt within a bacterium-tolerant virucide especially if sufficient disruption volumes are not achievable
10. Avoid changing conditions mid-experiment, unless that is the intention of an experiment
  - Report what medium is used during phage treatments, even if it is the same used for biofilm growth
  - Discuss why, and possible consequences, of any changes to media or conditions made during experiments
11. Characterization under multiple conditions toward improving the robustness of conclusions
  - Seek out alternative conditions for biofilm growth toward better representing in vivo conditions
  - Discuss limitations of conditions tested and possible alternatives that might also be tested
12. Keeping in vitro biofilms real
  - Describe conditions for biofilm growth and treatment that are thought to be present in situ or in vivo
  - Discuss how in vitro conditions used may differ from those thought to be found in situ or in vivo

^1^ Abbreviations: CFUs (colony-forming units), EPS (extracellular polymeric substance), MOI (multiplicity of infection), and PFUs (plaque-forming units).

## 2. Contrasting Biofilm Prevention, Control, and Removal

**Figure 1:**
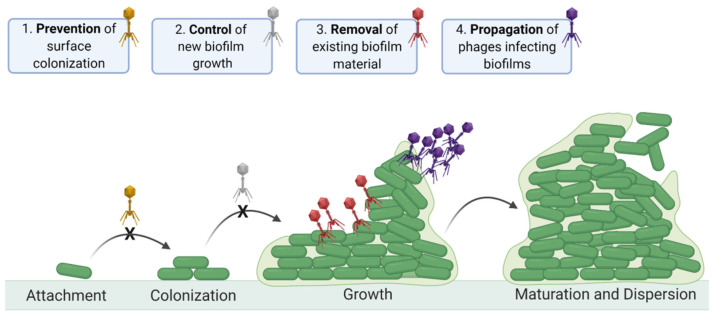
Different aspects of phage-biofilm interactions. Across the bottom: Biofilms begin with bacteria *attaching* to surfaces and to each other. This is followed by bacterial transition from planktonic to sessile lifestyles (*colonization*) and then to increases in biofilm bulk, i.e., as a consequence of a combination of biofilm-bacteria replication and extracellular matrix production (*growth*). Net growth ceases in association with biofilm *maturation*. Biofilms also can display various forms of cell *dispersion*, allowing for colonization of new surfaces. Across the top: (1) Phage *prevention* of bacterial surface colonization (mustard-colored virions), (2) phage-associated *control* of growth in biofilm bulk (gray-colored virions), and (3) phage-mediated *removal* of existing biofilm material (red-colored virions). In addition, (4) phages can produce new virions (*propagation*, purple-colored virions). This figure has been adapted from “Polymicrobial biofilm” by BioRender.com (2020) and retrieved from https://app.biorender.com/biorender-templates.

Various approaches to phage-biofilm in vitro experimentation can result in biofilm prevention, control, or removal (Figure 1). In this section, we distinguish these concepts, especially as it should be easier for phages to prevent biofilm initiation than to control the accumulation of new biofilm material, or to remove existing biofilm.

Biofilm *prevention* occurs prior to biofilm formation, especially with phages impacting still-planktonic bacterial cultures. Biofilm *control* specifically consists of interference by phages with the accumulation by biofilms of additional bacteria or matrix material. Control then is distinct from prevention in that it can occur only if biofilms are not yet fully mature, as we define ‘mature’ as a state in which *net* increases in biofilm presence (cells and matrix) is no longer occurring. Biofilm *removal* explicitly results in reductions in that amount of biofilm material present prior to encounter with phages.

Distinguishing biofilm control from biofilm removal can be a function not just of the timing of phage addition relative to stages of biofilm formation—with control seen only if biofilms are still growing—but also a function of what serves in experiments as negative-treatment controls. Specifically, it can be difficult to distinguish control of biofilm growth from removal of existing biofilm material unless phage anti-biofilm activities are compared with biofilm properties existing just prior to the start of phage treatments, that is, at ‘zero time points’ or ‘time zero’. Only if biofilm is removed will biofilm presence after phage treatment be reduced relative to biofilm presence at time zero, as we discuss further in Section 3.1. Note that blocking of biofilm growth without removing existing biofilm material also may be seen when employing bacteriostatic antibiotics as anti-biofilm agents [60].

## 3. Improving Phage-Biofilm In Vitro Experimentation

### 3.1. Distinguishing Control from Removal: Importance of Zero-Time-Point Determinations

**Figure 2:**
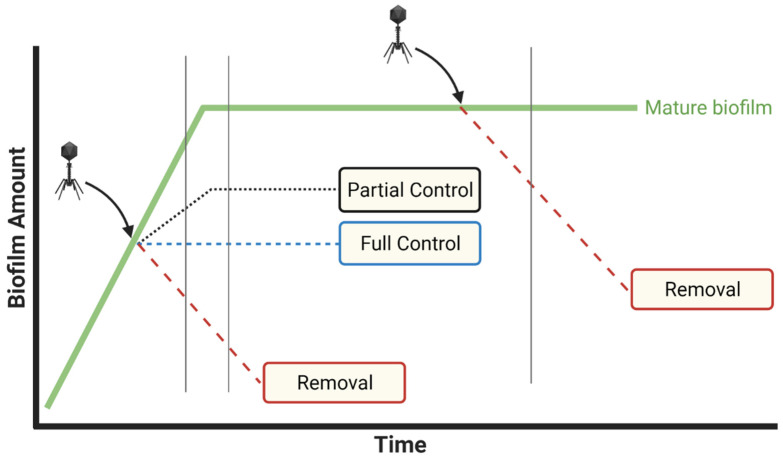
Control of biofilm growth vs. removal of existing biofilm. The extent of phage impact on biofilms can be contingent on when phages are added as well as what is used as a negative-treatment control. Toward illustration, two hypothetical experiments with different zero time points are presented: prior to biofilm maturation and after biofilm maturation (downward-pointing arrows). Mock treatment is shown as a solid-green curve ending in the “Mature biofilm” label. Negative controls consist of determinations of biofilm properties either at zero times (arrows) or post-treatment (corresponding to vertical gray lines). “Partial Control” indicates that biofilm growth might be possible even in phage presence, though less growth than with mock treatment. Created with BioRender.com.

Many phage-biofilm in vitro studies seem to focus on the control of new biofilm growth rather than on biofilm removal. Assessment of control may be emphasized particularly if (i) biofilms are still growing at the point of phage application and (ii) phage application to biofilms is compared only to mock-treatment controls. Dickey and Perrot [61] suggest, however, and we agree, that comparing the impact of anti-biofilm agents to zero-time-point biofilm properties is a more conservative approach. In particular, if we do not know how much biofilm was present at the point of phage application, then we have no means of determining to what degree phages have removed existing biofilm vs. only interfered with (i.e., controlled) the addition of new biofilm (Figure 2).

Given treatment of fully mature biofilms, then amounts of biofilm present just prior to phage application can indeed be equivalent to amounts present following mock treatment and subsequent incubation. Such equivalence, however, should be explicitly demonstrated rather than only assumed. This is because using mock treatments in combination with biofilms that are not fully mature at the start of treatments can bias phage impacts—to uncertain degrees—toward the as-noted control of biofilm growth rather than removal of existing biofilm.

Despite this utility of comparing biofilm properties to those present just prior to phage addition, it is still important to employ mock-treatment controls in experiments. This is because biofilm properties can change over the course of further incubation even without phage addition. For example, declines in biofilm biomass can spontaneously occur [62], which without mock-treatment controls could be mistaken for phage impact. Furthermore, studies that seek to model biofilm control but not removal should *not* be discounted. Authors, though, should be explicit in indicating that biofilm control is a study’s objective as well as whether phage-mediated biofilm removal has been explicitly measured in comparison to zero-time-point controls.

### 3.2. Knowledge of Phage Titers Is Needed for Interpretation and Reproducibility of Experiments

Outside of the laboratory, whether in the natural environment or during antibacterial treatments, the most readily available measures of culturable phage presence are phage titers, i.e., determinations of PFUs per mL [50,63,64,65,66]. Too often, however, phage doses are not provided in publications as titers, nor in a number of cases is a means of calculating phage doses as titers provided. This tendency seems to stem from a tradition that has developed in the phage literature of providing dosing information in terms of phage multiplicities of infection [67], i.e., MOIs, rather than explicitly as titers. This is problematic for a number of reasons, not least of which is because titer information is explicitly required both to repeat experiments and to translate experiments to applications outside of the laboratory. Also, phage adsorption kinetics are studied and understood in terms of phage titers rather than MOIs [68,69,70].

In addition, if MOIs are held constant, then phage titers will change depending on bacterial numbers. Rates that phages find bacteria consequently can change dramatically if MOI is held constant but bacterial densities are not. For example, when bacteria are present at densities of 10^8^/mL, then with an MOI of 1, and therefore a titer of 10^8^/mL, the mean free time of a bacterium, that is, average time until a bacterium is adsorbed [71], can be approximately 5 min. At a bacterial density of 10^5^/mL, however, with an MOI 1 and therefore a titer of 10^5^/mL, we would expect that same bacterial mean free time to be approximately 5000 min, or greater than 2 days.

MOIs can be translated into titer information by readers only if it is obvious whether reported bacterial numbers are those present at the point of phage addition or instead are amounts present at the point of initiation of biofilm cultures, numbers which can differ substantially. Dosing in some cases is provided also in terms of number of phages supplied per environment, e.g., phages per microtiter plate well, rather than as titers. In this case, titer information can only be obtained from MOI information if culture volumes are also reported along with bacterial densities. Unfortunately, however, volume information too often is not provided. To avoid these various issues, phage titers as found in situ following phage addition, whether as measured or as anticipated, should be unambiguously described in studies (Box 2).

Box 2 **Describing in situ phage titers.**

Dosing measured in phage titers, in combination with dosed volumes (e.g., 20 μL), recipient or resulting volumes (e.g., 180 or 200 μL), and if possible numbers of recipient bacteria, should represent minimum dosing descriptions. Thus, for example,

Biofilms consisting of 2.0 × 10^7^ CFU/well were washed and 2.0 × 10^7^ PFU suspended in 200 μL of broth were added, resulting in a final well volume of 200 μL, an initial well titer of 10^8^ PFU/mL, and a MOI of 1.

Somewhat typically, however, one sees instead the equivalent of,

Phages were added to biofilms at a multiplicity of 1.

The latter does not supply initial in situ phage titer information, time-zero CFU numbers, or necessarily whether the multiplicity is based on numbers of CFUs present at the point of phage addition or instead numbers of CFUs supplied to initiate biofilms. The result of such brevity in descriptions of methods can be a literature that is somewhat impenetrable pharmacologically, ecologically, and in terms of reproducibility.

**Figure 3:**
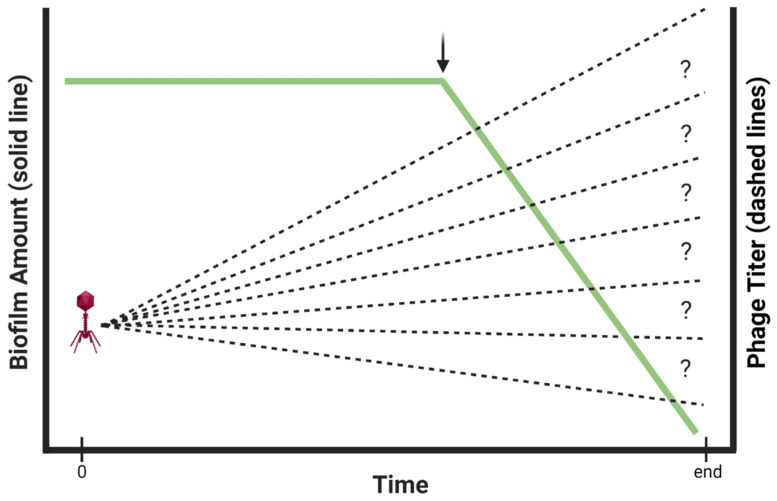
What phage titer ultimately is required to clear biofilms? Solid line: biofilm presence. Dashed lines: phage titer and increase due to in situ phage propagation, as starting with an MOI of somewhat less than 1. Scales for *y* axes to the left and to the right are not necessarily equivalent. Illustrated is the idea that there is little possibility of understanding phage-bacterial population dynamics without quantifying phage titers over time in combination with at what point in time biofilm amounts start to be reduced (arrow). Toward increasing clarity, depiction of synchronous phage replication, e.g., as seen in Figure 1 of [80], is intentionally not provided in the figure. Created with BioRender.com.

Indications during experiments of temporal *changes* in phage titers often are also not provided in studies of phage-biofilm interactions. As a consequence, there can be no means of knowing how long initial phage doses are retained, whether phage numbers decline over time, or instead whether phage numbers increase in the course of phage in situ propagation (Figure 3). In clinical settings, declines in concentrations of antibacterials found in association with targeted bacteria would suggest a need for drug re-application, and in clinical practice phages often are delivered not as only a single dose per treatment [72,73,74,75,76,77,78,79]. Thus, measuring phage titers over time, minimally such as at time zero in combination with end-point determinations, can be relevant to translating phage treatments of biofilm from in vitro or in vivo experimentation to clinical settings, or otherwise toward better appreciation of the ecology of phage-biofilm interactions. Alternatively, absence of knowledge of how phage titers change over the course of experiments can result in a reduced mechanistic understanding of ensuing phage therapy successes or failures, or reduced understanding of the ecological dynamics of phage-biofilm interactions.

### 3.3. The Conceptual Problem of per-Area Rather Than Titer-Based Dosing

A convenient approach to describing phage application to biofilms is on a per-area basis. In terms of increasing our understanding phage-biofilm interactions, however, such per-area dosing is less helpful than standard titer-volume dosing descriptions. The issue is that in situ phage titers represent key dosing starting points, since the likelihood that a targeted bacterium will become phage infected is directly proportional to phage titer [68,69,70]. Thus, phage dosing that is indicated on a per-area basis, but which does not include in situ titer information (Box 2), provides little indication of the potential kinetics of phage adsorption.

It is possible to make the following counter argument: Given sufficiently high densities of target bacteria in combination with sufficiently small volumes of applied phages, then approximately all applied phages in fact may adsorb. For example, if 10^6^ phages were applied per cm^2^ within a very small volume (e.g., a few μL), then we might expect 10^6^ phage infections at the start of treatments—assuming that all phages successfully find bacteria to adsorb, that is, rather than being lost to run-off from the surface, and that infections predominantly are initiated with single rather than multiple virion adsorptions. It is questionable, however, how such a strategy of dosing, one of phage numbers per unit area of biofilm, might be applied to actual use outside of the laboratory. That is, applied phage titers and volumes certainly would still be key dosing parameters, even if these are to be adjusted depending on the total area of the to-be treated surface. Phage titer and phage volume information therefore should always be included in descriptions of phage dosing [81] (Box 2), even if one also includes descriptions of ratios of phage doses to treated areas.

### 3.4. Overlying Fluid Small-Volume Effects

The more we require that phages propagate, especially by dosing phages at MOIs of less than 1, then the more we need to be assured that this phage propagation is realistic relative to real-world circumstances. Ideally, during biofilm experiments, phages therefore should be propagating more or less exclusively on those bacteria that are associated with biofilms. Buildup of phage numbers during experiments, however, is not necessarily always driven by phage infection of biofilm bacteria but instead can be due to phage infection of planktonic bacteria as well.

Even if we wash away planktonic bacteria prior to phage application, these bacteria nevertheless can build up in the vicinity of biofilms, re: dispersion (Figure 1). This build up would be especially so given small, fixed volumes surrounding biofilms and low starting phage titers. As a result, it can be possible for planktonic bacteria to support phage propagation to higher titers than may be achieved in the absence of planktonic bacteria, resulting in a greater phage impact on biofilms than phages propagating on biofilm bacteria alone might be able to achieve. This, in turn, could lead to misleading conclusions that application of relatively low phage titers can be as effective for anti-biofilm treatments as application of higher phage titers. Perhaps flow conditions [82,83]—starting with static phage adsorption periods but followed by continuous removal of planktonic bacteria—therefore would be a better choice than static-based biofilm cultivation when exploring the impact of low phage titers on bacterial biofilms, since in constant flow environment there is less chance for planktonic bacteria to build up around biofilms.

Phage propagation, whether in association with biofilms or instead upon infecting planktonic bacteria, equivalently can lead to increases in phage titers not only within biofilms but in overlying fluids as well. Building up of phage titers in overlying fluids, however, is not necessarily representative of real-world circumstances, particularly if overlying volumes outside of the laboratory are large, e.g., streams, ponds, lakes, or even gastrointestinal tracts. Volumes during biofilm experiments in the laboratory by contrast can be small, e.g., 100 or 200 µL within 96-well microtiter plates, and in some cases subject to mixing. This could drive unnaturally greater, faster, or more widespread phage impact on biofilm bacteria since when volumes are much larger, then phages are more likely to disperse away from biofilms (Box 3).

### 3.5. Dosing with Insufficient Phage Titers?

**Figure 4:**
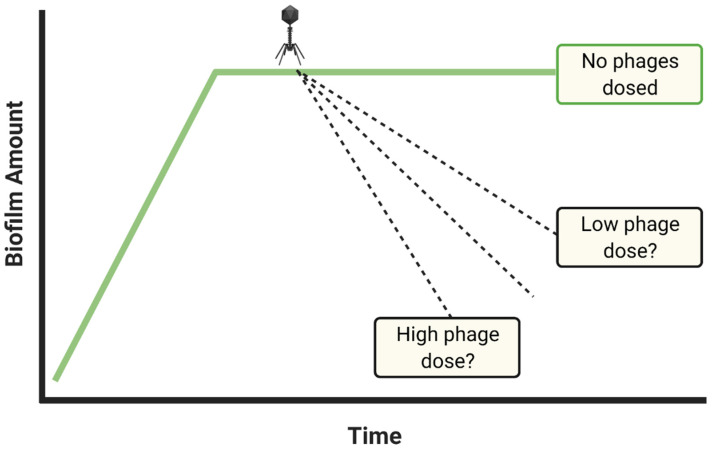
Possible impacts of different phage titers on biofilms. Pharmacologically, it is typical that the application of greater drug amounts will result in greater impacts on target tissues. Dosing with greater numbers of phages, as added in the figure at the phage icon, therefore should generally be attempted toward enhancing anti-biofilm activities, particularly when desired anti-biofilm activity is not otherwise achieved. This should especially be rather than employing as maximum doses phage MOIs of approximately 1 or lower. The middle dashed line, unlabeled, is meant to describe the results of some in-between phage dose. Created with BioRender.com.

There are two major reasons for poor phage anti-biofilm activity, and these are either not employing the right phage or instead not dosing with sufficient numbers of phages. The most easily implemented means of attempting to improve upon treatment results that are unsatisfactory is to apply more phages (Figure 4), and this is rather than to first switch phages. Increasing dosing per experiment ideally should continue until it can be demonstrated that it is *not* the number of phages that are being supplied that underlies insufficiencies in anti-biofilm efficacy. If anti-biofilm efficacy is still inadequate, then serious consideration should be given to testing new phages as anti-biofilm agents, or implementation of additional strategies aimed at increasing biofilm susceptibility to phages, such as multiple dosing, applying additional phage types, or co-treating with additional antibacterial agents such as antibiotics.

Box 3 **A small-volume argument for applying higher rather than lower phage titers to in vitro biofilms.**

Consider as an ecological thought experiment a very large biofilm, e.g., many cm^2^ in area, and its encounter with only a single phage. In this case, spreading of resulting phage progeny can occur either solely in association with the biofilm, as equivalent to phage plaque propagation [44,84,85], or instead with these phages released from the biofilm and spreading through overlying fluid as well. In the latter case, if overlying volumes are small enough then initiation of phage infections over multiple points on the targeted biofilm by these now planktonic virions could be more likely than had those virions remained within their source biofilm or had diffused outward into a much larger volume. The result could be an artificial acceleration of the overall phage impact. Small experimental volumes thus could aid not just phage propagation, i.e., given infecting of planktonic along with biofilm bacteria, but phage dissemination within the vicinity of biofilms as well.

Note that in either case, at least in terms of phage treatments of in vitro biofilms, we can avoid these complications by starting with relatively high phage numbers, e.g., 10^7^ PFU/mL or ideally [75] even higher. That is, when phages are applied to biofilms in higher numbers, then there should be less concern about the buildup of phage numbers around biofilms by means other than due to dosing or due to phage propagation within biofilms. Application of higher rather than lower phage titers—to the extent that this limits the degree of phage propagation required to control or remove biofilm material—should also better help to address the question posed in Figure 3, i.e., what phage titers ultimately are required to clear biofilms?

Application of higher phage densities, e.g., 10^8^ or 10^9^ PFU/mL, ideally would be built into all phage anti-biofilm studies, and especially if desired levels of biofilm removal are not otherwise achieved. Note that this issue is generalizable to that of Casadevall and Fang’s [59] statement that, “Good laboratory practices include… dose-response studies…” One caveat to this point of a possible utility to testing dosing with higher phage numbers, however, may occur if biofilms are being treated with phage cocktails rather than with individual phage types. Specifically, it is possible that upon application of phage cocktails, infection of the same bacteria by different phages could interfere with each other’s bacteriolytic or phage production activities [86,87,88,89,90]. That this possibility exists, however, should not be seen as reason to avoid testing higher phage doses.

### 3.6. Insufficient Numbers of Time Points?

**Figure 5:**
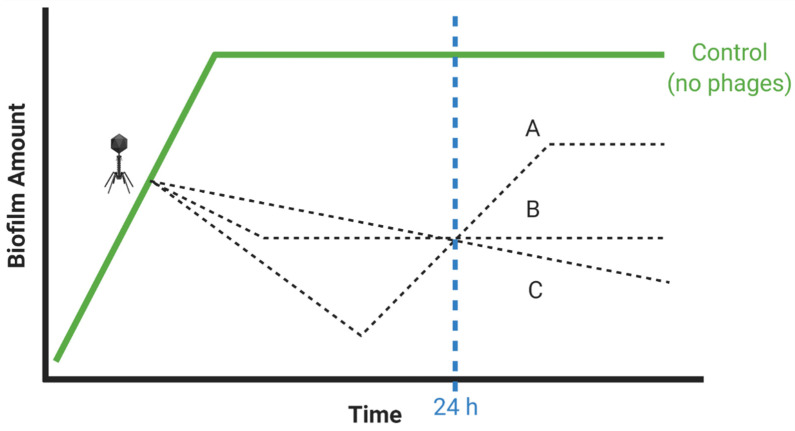
End-point analyses tend to overly simplify population dynamics. Many studies take their first and often only time point at 24 h following phage application. Though it is possible that bacteria killing or biofilm disruption is still ongoing after this length of time (C), it also is possible that bacterial populations or biofilm presence instead is recovering (A), or neither declining nor recovering (B). Possible underlying mechanisms for the shape of the presented curves are: (A) Phage-mediated reductions in biofilm presence followed by grow back of less biofilm-forming-capable, phage-resistant mutants. (B) Incomplete phage-mediated reductions in biofilm presence. (C) Ongoing but slow phage-mediated reductions in biofilm presence. Created with BioRender.com.

From Casadevall and Fang [59], “Good laboratory practices include… determination of time courses…” Particularly, it often is not easy to appreciate the dynamics of phage impact on bacterial cultures without doing some kinetic analyses, as can be accomplished by taking multiple, well-separated time points, or by exploring different treatment durations in separate experiments, e.g., 12 vs. 24 h. Such approaches can be useful in terms of characterizing rates of phage impact on biofilms as well as rates of phage propagation, the latter relevant especially if such propagation is required for phages to have a substantial impact on biofilms, i.e., toward active treatments [71]. Kinetic determinations also can document biofilm grow back over the course of treatments, which would seem to be the case when more biofilm is present after longer vs. shorter treatment periods [91,92]. See Figure 5 for illustration of treatment outcomes that may be distinguished by taking multiple rather than only single time points.

### 3.7. Enzymatic Biofilm Matrix Disruption

Phages can possess more than one mechanism of biofilm removal. First is the infection and lysis of bacteria, which is our primary emphasis here. In addition, phages can encode biofilm matrix-, LPS-, or capsule-degrading enzymes. These enzymes are often referred to as extracellular polymeric substance (EPS) depolymerases [93,94], and phage-encoded EPS depolymerases typically are components of virion particles [95]. Phage carriage of EPS depolymerases is important to the interpretation of the impact of phages on biofilms as these enzymes can disrupt biofilms (Box 4) without associated phage-induced bacterial lysis [96,97]. This can be the case given dosing with relatively large numbers of replication-incompetent phages or phage dosing in combination with antibiotics known to interfere with the production of new virions during phage infections [98].

Typically, phages encoding EPS depolymerases will generate plaques that are surrounded by halos that increase in size over extended times of incubation while growing on indicator bacteria that produce enzymatically susceptible EPS [99,100,101,102]. Thus, it is helpful toward interpretation of the consequences of phage addition to biofilms for authors to monitor halo formation during phage plaquing and then to report plus or minus halo presence in publications. Notwithstanding that exhortation, usually authors appear to be highly motivated to mention this, though given the typically high specificity of EPS depolymerases, it is important that halo presence be confirmed for all experimentally targeted bacterial strains.

Box 4 **EPS depolymerase impacts on bacterial biofilms.**

The enzymatic activity of EPS depolymerases can prevent biofilm formation [102] or break down biofilm structure [103,104,105,106,107], releasing bacteria and biomass directly. EPS depolymerases might also increase biofilm susceptibility to disruption upon washing, where washing or rinsing is typically done prior to in vitro-grown biofilm characterization. EPS depolymerases also can form tunnels through biofilm matrix without substantially decreasing biofilm biomass, but improving diffusion through biofilm structure [103,108]. Latka and Drulis-Kawa [107] found that EPS depolymerase application increased crystal violet staining while not reducing numbers of biofilm CFUs. EPS depolymerase action also may augment the potential for phage infection and associated bacterial lysis, e.g., by making bacteria more available to phage adsorption [89,109]. A related issue is the question of to what extent reductions in cell counts given presence of phage EPS depolymerase activity are due to decreases in the viability of biofilm bacteria vs. conversion of attached bacteria to planktonic cells, outcomes which may be viewed very differently from both ecological and therapeutic perspectives.

### 3.8. Limitations of Biofilm Biomass Determinations

Methods used for determining biofilm characteristics can be differentiated into those that are primarily physical or chemical vs. primarily biological or microbiological, the latter including especially CFU determinations (next section). Among physiochemical approaches, dye-based methods are most commonly used [110], such as staining using crystal violet [111], dimethyl methylene blue [112,113,114], or SYTO 9 [107,115,116]. In addition are combination physiochemical and biological approaches using stains that indicate bacterial viability such as resazurin [117,118] and soluble tetrazolium dyes [110,119,120,121]. Especially crystal violet staining is used in determining the phage impact on bacterial biofilms, particularly in 96-well microtiter plate formats [108,122,123,124]. This allows total biofilm biomass quantification [125,126]. Nonetheless, there can be a number of issues with using primarily biomass determinations to assess phage impact on biofilms. For instance, often biofilm staining such as with crystal violet results in large variations in measurements between replicas [110], though this can be improved [127].

A second issue is that differences in biofilm assessment can result in different perceived outcomes. For example, whereas phages may substantially impact numbers of viable bacteria, they may not similarly remove EPS, or vice versa. Phages may not even fully remove otherwise lysed or killed bacteria, which thereby could continue to contribute to the amount of biomass detected. Danis-Wlodarczyk et al. [128], for example, noted a lack of biofilm biomass reduction despite an approximately 1-log CFU reduction.

A third issue, one related to the second, is a consequence of bacterial numbers in general needing to be calibrated against *estimations* of bacterial numbers, such as from biofilm biomass determinations, before those estimations may be used as surrogates to CFU determinations. Thus, if a goal of phage application to biofilms is reductions in or at least measurements of changes in biofilm bacterial counts, then use of biomass as a primary measurement by necessity will require generation and use of calibration curves. Many phage studies that rely upon biomass reductions as a measure of phage impact, however, do not provide calibration curves associating different amounts of biofilm biomass with different numbers of viable biofilm bacteria. Indeed, it is not obvious even how such phage-mediated biofilm-removal, biomass-to-CFU calibration curves might be accomplished.

Lastly, phage-mediated determinations of reductions in biofilm biomass often are presented in publications using linear rather than logarithmic scales, i.e., 0, 10 20… 80, 90, 100% rather than, e.g., 10^−4^, 10^−3^, 10^−2^, 10^−1^, and 10^0^. Particularly for therapeutic impacts of phages on biofilm bacteria to be substantial—such as reductions in excess of 100-fold in biofilm presence—those impacts would need to be graphed using log-amounts-of-biofilm scales to be distinguishable from lesser reductions. That is, reductions of only 10-fold (10^−1^) in biofilm biomass, or even only 100-fold (10^−2^), with the latter barely registering above zero on a linear scale, should not be viewed as substantial from a therapeutic standpoint, and particularly not when biofilm eradication is a goal.

### 3.9. Colony Count Complications

**Figure 6:**
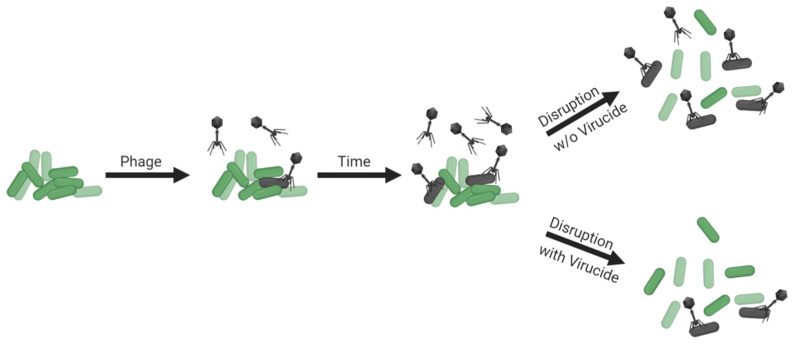
Illustration of greater susceptibility of bacteria to phage adsorption given biofilm disruption without virucides present (or without sufficient dilution; **top**) or with virucides present (or with sufficient dilution; **bottom**). Bacteria adsorbed by phages are indicated to the right. Created with BioRender.com.

Though non-culture-based approaches to estimating bacterial viability within biofilms exist [107,108,129,130,131,132,133,134] (and see the previous section for additional references), determination of numbers of biofilm-associated CFUs remains an important means of assessing impacts of phages on biofilm bacteria. Determining biofilm CFU counts, though, requires first that bacteria are separated from each other. The structure of still-intact biofilms, however, may reduce phage access to constituting bacteria, such as due to bacteria being buried beneath other bacteria [84,135,136,137] or due to biofilm matrix serving as a virion-diffusion barrier [138,139]. As a consequence, disrupting biofilm structure to separate individual cells for enumeration could make those bacteria more susceptible to phage adsorption (illustrated in Figure 6, top panel), and bacteria released by biofilm disruption indeed become more susceptible to adsorption to phages that are added to cultures following that disruption [139,140]. To accurately assess numbers of biofilm-associated CFUs, it therefore can be crucial to prevent phages from adsorbing bacteria following biofilm disruption, and this concern could be particularly relevant if those phages are present within biofilms in relatively high numbers.

Prevention of phage adsorption during biofilm disruption, or during bacterial plating more generally [141], can be accomplished by first inactivating phages and/or by substantially diluting biofilms in the course of their disruption (Figure 5, bottom panel). If these approaches are not employed, then additional phage infections can occur, with CFU numbers thereby lowered from what were present prior to the enumeration step. A utility for virion inactivation prior to biofilm disruption for enumeration, however, is not necessarily always indicated in biofilm-characterization protocols [142]. There nevertheless are several virucides analyzed in the literature which are known to inactivate free virions but not phage-uninfected bacteria. These include ferrous sulfate and extract of rinds of *Punica granatum* (pomegranate), leaves or flowers of *Viburnum plicatum* (Japanese snowball), leaves of *Camellia sinensis* (tea), leaves of *Acer saccharum* (sugar maple), or the pits of *Phoenix dactylifera* (date palm) [143,144,145,146,147,148,149,150].

Though inactivation of bacteria by phages during enumeration can be a concern, it is one that is not routinely explored during phage-biofilm in vitro experimentation. Ideally, if performing experiments without using virucides is preferred, then levels of dilution during biofilm disruption would be assessed for their potential to protect bacteria from associated phages. Virucides would then be employed if levels of dilution prior to that disruption are found to be inadequate—that is, insufficient to protect bacteria from associated phages—and if for technical reasons pre-disruption dilutions cannot be further increased.

### 3.10. Avoid Changing Conditions Mid-Experiment, Unless That Is the Intention of an Experiment

In some phage-biofilm studies, conditions appear to change in the middle of experiments, typically without explicit justification. Especially of concern are modifying bacterial growth conditions such as by qualitatively switching what fluids overlay biofilms in the course of adding phages. Changing conditions certainly can be legitimate if the point of an experiment is to observe how these changes might affect phage impact in comparison to appropriate controls, e.g., phages applied during phage therapy generally will be first suspended in something other than bodily fluids. Changing conditions mid-experiment, however, should not be done without justification, and certainly should not be done without explicit indication. Changing conditions, that is, might affect biofilm integrity or phage infection dynamics, thereby making biofilms more or instead less susceptible to added phages.

A typical condition change is from broth medium as the biofilm overlaying fluid instead to buffers. Generally, adequate host metabolism to support phage infections requires providing bacteria with sufficient energy supplies. Phage application to biofilms in a manner that limits the access of bacteria to energy supplies in particular will tend to limit the potential for phages to infect productively or limit the potential for phages to infect lytically once they have adsorbed target bacteria [151]. In addition, decreases in bacterial numbers or biofilm biomass can occur solely due to starvation [152,153,154,155]. As a number of studies appear to be using buffer- or saline-suspended phages as treatments, in many cases without clearly indicating whether or not this is the case, it seems prudent for authors to unambiguously highlight what it is that phages have been suspended in during treatments, even if that media is the same as what biofilms were grown in.

### 3.11. Characterization under Multiple Conditions toward Improving Robustness of Conclusions

Biofilm characteristics, and presumably phage-biofilm interactions as well, can vary as a function of medium, substratum, and other aspects of growth conditions. Medium composition, for example, can have large influences on biofilm properties, something that is often overlooked in biofilm studies [127]. Additionally, changing medium composition can alter the staining patterns of the dye-based methods often used in phage anti-biofilm evaluation studies (Section 3.8). Ideally, though, in vitro models of biofilms should substantially mimic those conditions that are found in vivo or in situ, making concerns about such divergence of biofilm properties with conditions moot. If substantial mimicry of expected in situ conditions is not the case, however, then reliance on only one set of conditions for biofilm growth and treatment—such as only one type of substratum material, or only a single type of growth medium, or indeed phage treatment of only a single bacterial strain (or single-species vs. multiple-species biofilms)—could interfere with recognition of variation in phage susceptibility.

Discovering excessive variation under different experimental conditions should serve as a warning for a greater need to mimic in situ conditions in vitro (next section). Alternatively, again from Casadevall and Fang [59]: “Results that remain robust despite variance in experimental conditions are more likely to be valid.” All of these statements point to a utility to not limiting analyses of phage-biofilm interactions to only a single set of conditions, unless the point of a study explicitly is to study those specific conditions without comparison with other conditions.

### 3.12. Keeping In Vitro Biofilms Real

Beyond experimental consistency (previous section), a challenge for phage-biofilm studies is the establishment of model systems and conditions that reasonably mimic those found in situ or in vivo, whether in animals (e.g., mouse or pig) or in other natural settings (e.g., a pond). For controlled biofilm studies, such mimicry can be accomplished in situ either by studying biofilms as they develop under natural conditions [156], as following experimental inoculation into environments with biofilm-forming bacteria, or instead by inserting in vitro-grown biofilms into those environments, e.g., such as biofilm-harboring plastic into rats [157]. In addition, either naturally occurring substrata (e.g., exposed tissues, rocks) or artificial substrata (e.g., sterilized wafers or implants) may be employed, also in situ.

Phage addition can be accomplished either within the environment in which a biofilm has developed or instead upon removal of in situ-grown biofilms to in vitro environments, thus, ex situ. Examples of this latter approach include root-canal [158], porcine-skin [159,160], human-urine [161], or tissue-culture monolayer [162] models. Studying biofilms removed from in situ environments to in vitro ones could, however, at least in principle result in creating small-volume effects (Section 3.4) as well as changes in the composition of overlying media (Section 3.10). On the other hand, they also could allow comparison between in vitro- and in situ-grown biofilms, that is, as a test of how realistic the former are as approximations of the latter.

In addition to standard techniques used for the characterization of intact biofilms (e.g., confocal or electron microscopy), so too can phage impact be used as a measure of the properties of in situ- vs. in vitro-grown biofilms. That is, changes in phage titers or degrees of phage-mediated removal of biofilms may be viewed not just from a perspective of determining phage characteristics but also from a perspective of comparing biofilm characteristics, e.g., such as in terms of phage resistance.

Even if conditions are well matched between in situ and in vitro conditions, there can still be differences in biofilm properties that are a function simply of the duration of biofilm growth prior to phage application [163]. Indeed, this can occur in situ as well, perhaps most notably with distinctions in the characteristics of acute vs. chronic bacterial infections, which can also be a challenge to distinguish in terms of in vivo infection models [30,75]. Above all, we caution against assumptions of equivalence between in vitro-grown biofilms and those grown in situ, as well as in situ vs. ex situ phage treatments, unless such equivalence has been rigorously demonstrated.

## 4. Conclusions

In vitro systems provide a relative ease of experimentation that is crucial for developing a robust understanding of the biology of phage-biofilm interactions as well as a means by which approaches to the treatment of biofilms using phages may be improved upon. At the same time, in vitro experiments should serve only as models for more elaborate in situ experimentation, with in situ and especially in vivo approaches often more expensive, more time consuming, and more limited in terms of what questions may be addressed. Here, we have explored how results derived from in vitro models of phage-biofilm interactions may be compromised by various, often common laboratory practices (see Box 1 for partial summary). Our goal certainly is not to discourage further in vitro experimentation but rather to encourage development of better approaches to these studies, particularly with greater awareness of when such explorations can become inadvertently misleading.

## Data Availability

Not applicable.
